# Sea cucumber grazing linked to enrichment of anaerobic microbial metabolisms in coral reef sediments

**DOI:** 10.1093/ismejo/wraf088

**Published:** 2025-05-03

**Authors:** Andrew J Maritan, Cody S Clements, Zoe A Pratte, Mark E Hay, Frank J Stewart

**Affiliations:** Department of Microbiology & Cell Biology, Montana State University, Bozeman, MT 59717, United States; School of Biological Sciences and Center for Microbial Dynamics and Infection, Georgia Institute of Technology, Atlanta, GA 30332, United States; Department of Microbiology & Cell Biology, Montana State University, Bozeman, MT 59717, United States; School of Biological Sciences and Center for Microbial Dynamics and Infection, Georgia Institute of Technology, Atlanta, GA 30332, United States; Department of Microbiology & Cell Biology, Montana State University, Bozeman, MT 59717, United States; School of Biological Sciences and Center for Microbial Dynamics and Infection, Georgia Institute of Technology, Atlanta, GA 30332, United States

**Keywords:** marine microbiome, community ecology, metagenomics, oxygen, conservation, facilitation

## Abstract

Sea cucumbers have been overharvested world-wide, making assessments of their ecological effects challenging, but recent research demonstrated that sea cucumbers increased coral survival via disease suppression and were therefore important for facilitating reef health. The mechanisms underpinning the sea cucumber-coral interaction are not well understood but are likely mediated through sea cucumber grazing of microbes from reef sediments. We explored how sea cucumber grazing alters the sediment microbiome by leveraging a healthy sea cucumber population on a reef in French Polynesia. We used quantitative PCR, 16S rRNA gene sequencing, and shotgun metagenomics to compare the sediment microbiome in cages placed *in situ* with or without sea cucumbers. We hypothesized that grazing would lower microbial biomass, change sediment microbiome composition, and deplete sediment metagenomes of anaerobic metabolisms, likely due to aeration of the sediments. Sea cucumber grazing resulted in a 75% reduction in 16S rRNA gene abundances and reshaped microbiome composition, causing a significant decrease of cyanobacteria and other phototrophs relative to ungrazed sediments. Grazing also resulted in a depletion of genes associated with cyanotoxin synthesis, suggesting a potential link to coral health. In contrast to expectations, grazed sediment metagenomes were enriched with marker genes of diverse anaerobic or microaerophilic metabolisms, including those encoding high oxygen affinity cytochrome oxidases. This enrichment differs from patterns linked to other bioturbating invertebrates. We hypothesize that grazing enriches anaerobic processes in sediment microbiomes through removal of oxygen-producing autotrophs, fecal deposition of sea cucumber gut-associated anaerobes, or modification of sediment diffusibility. These results suggest that sea cucumber harvesting influences biogeochemical processes in reef sediments, potentially mediating coral survival by altering the sediment microbiome and its production of coral-influencing metabolites.

## Introduction

Corals are the foundation species of tropical reef ecosystems but have rapidly declined worldwide from diverse and compounding stressors including disease, pollution, overfishing, and ocean warming and acidification [1–3]. Given that many human-mediated stressors are predicted to increase in frequency and severity, there is increasing interest in leveraging biotic interactions to increase coral survival and provide more tractable and effective management options [4]. For example, sustaining healthy and diverse communities of herbivorous fishes can positively affect corals by removing harmful seaweeds [5]. However, the coral-seaweed-fish relationship can change from exerting positive to negative effects on corals depending on environmental conditions [6] or the presence-absence of third-party consumers, e.g. sea stars that eat corals but cannot find corals under high seaweed cover [7]. The diversity of reef communities suggests that coral health could be influenced by many other multi-species interactions that remain largely uncharacterized.

Sea cucumber-coral interactions have recently been identified as important for coral health and potential targets for coral reef management [8, 9]. Sea cucumbers (Class Holothuroidea) are important cleaners of reef sediments by removing sediment organic material and microbes during feeding [10]. These grazers were once commonplace on reefs but have been globally overharvested for decades to centuries, with recent harvest estimates of more than 1 billion individuals per year [11]. This overharvesting may have serious overlooked implications for reefs if sea cucumbers influence coral health, either directly or indirectly. In some less populated areas of the world, sea cucumber populations remain strong, either because they are inaccessible [9] or undesirable for human consumption [11] and thus can be used in manipulative experiments to test the sea cucumber-coral relationship. Recent such experiments on reefs of Mo‘orea, French Polynesia and Palmyra Atoll revealed that whole colony mortality of reef-building acroporid corals increased up to 15 times in the absence versus presence of sea cucumbers, with tissue death consistently beginning at the sediment-coral interface and progressing up the coral branch [9]. When corals were separated from direct contact with the sediments (e.g. by having turf algae at their bases to act as a living barrier to sediment contact), colony tissue death decreased by 94–100%. This prior work also confirmed that sea cucumber grazing altered the taxonomic composition of sediment microbiomes and that coral-associated microbiomes differed between diseased and healthy corals [9]. Together, these results link coral mortality to contact with sediments and identify sea cucumber grazing as a significant influence on coral mortality, potentially by affecting sediment microbiome composition and function.

Prior study of the sea cucumber-sediment-coral relationship did not identify a pathogen(s) linking coral disease to the altered sediment microbiome, raising the question: how exactly do cucumber-driven changes to the sediment microbiome influence coral health? We hypothesized that in the absence of sea cucumber grazing, sediment microbiomes negatively affect corals through diverse metabolic mechanisms. Such mechanisms may influence coral exposure to anoxia (from the absence of mechanical aeration of sediments from sea cucumber grazing; [10]), acidity, nutrients, or harmful chemicals. The latter may include toxic products of metabolism, such as sulfide generated by sulfate reduction in sediment biofilms [12], or toxins from pathogens or benthic cyanobacteria [13]. The effect of such products on corals, as well as the potential for sediment oxygen depletion, will presumably vary with microbial load, which is expected to be lower in sediments grazed by sea cucumbers due to microbial consumption [10] but was not measured in prior work. It is also possible that grazing removes coral-relevant microbes from sediment during passage through the sea cucumber GI tract or enriches coral-relevant microbes in sediment upon deposition of feces.

To explore potential mechanisms by which sea cucumber grazing affects sediment microbiomes and biogeochemical processes, we quantified (i) microbiome abundance, taxonomic composition, and metabolic potential in sediments exposed to or protected from sea cucumber grazing, and (ii) microbiome composition dynamics during passage through the sea cucumber gut. We used cages with and without sea cucumbers to experimentally simulate sea cucumber presence and absence *in situ* on a reef of Mo‘orea, French Polynesia. Cages without sea cucumbers simulated conditions following overharvest of sea cucumbers. Using this framework, we sampled ungrazed sediment (sed w/o sea cucumbers), grazed sediment (sed w/), and partially digested sediment from the sea cucumber GI tract. We used DNA from these samples to estimate prokaryotic microbial load via quantitative PCR (qPCR) counts of 16S rRNA genes and microbial taxonomic and functional gene composition via 16S rRNA gene and shotgun metagenomic sequencing. The results confirm that grazing significantly reshapes the biochemical landscape of the sediment microbiome, particularly affecting the potential for oxygen utilization and cyanotoxin production.

## Materials and methods

### Cage experiments and sample collection

**Figure 1:**
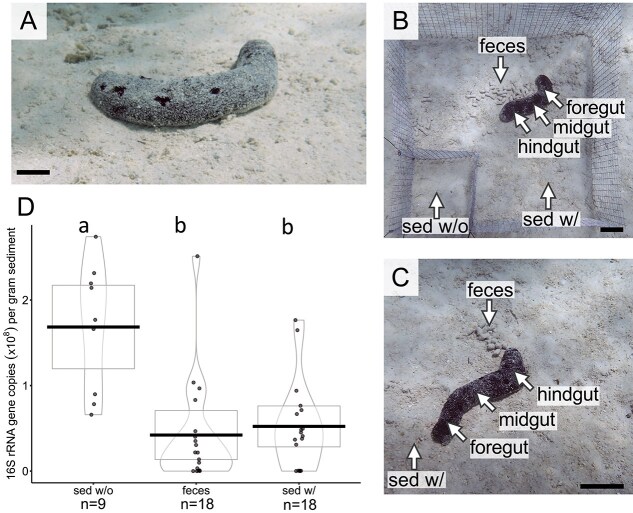
Sediment microbial load decreases in the presence of the sea cucumber *Holothuria atra*. (A) Representative *H. atra* individual at the field site in Mo‘orea, French Polynesia. Scale bar 1 cm, photo credit: A.J. Maritan. (B, C) experimental design for testing effects of sea cucumber grazing on reef sediment microbiomes (diagram not to scale for clarity). (B) Wire mesh cages (n = 9; 50 × 50 × 14.4 cm) with inset cages (14.4 × 14.4 × 14.4 cm) were used to exclude grazing for 6 days and then to enclose a sea cucumber for 20.5–22 h before collection of ungrazed sediment (sed w/o), grazed sediment (sed w/), and fecal samples. Foregut, midgut, and hindgut samples were also dissected from collected individuals. (C) Free-roaming (outside cages) sea cucumbers (n = 9) were collected concurrently with caged sea cucumbers and used for sampling of sed w/, foregut, midgut, hindgut, and fecal microbiomes to assess possible artifacts of caging. Scale bar 5 cm, photo credit: C.S. Clements. (D) Quantitative PCR (qPCR) of 16S rRNA gene copies normalized to grams of sediment used in DNA extraction, presented as violin plots (mean as estimate of center; box bounded by 95% confidence interval; density estimator used to draw violin; observations as individual points). Microbiome samples from caged (B) versus free-ranging sea cucumbers (C) did not differ in qPCR microbial abundance (*P >* .811; Supplementary Fig. 4A) so those samples are pooled for this figure. Dissimilar letters indicate *P <* .0001 in pairwise comparison between groups, whereas sharing of any letter indicates *P >* .05 (*P* values were obtained from linear mixed effects model; Tukey–Kramer HSD).

We examined sediment microbiomes from cages with and without *Holothuria atra* sea cucumbers and from areas external to the cages (Fig. 1A, B, C). For cage experiments, we erected ten 50 × 50 × 14.4 cm (LWH) cages (1 cm mesh size) on 11 June 2021 on sandy surfaces at a depth of ~2 m in the north shore lagoon of Mo‘orea, French Polynesia (−17.489340, −149.885769). This site supports a population of *H. atra* at an average density of 7.1/m^2^ [8]. For 6 days, sea cucumbers were excluded from cages to allow sediment microbiomes to develop without sea cucumber grazing. On day 7, a second smaller cage (14.4 × 14.4 × 14.4 cm) was inset in one corner of each larger cage, and one *H. atra* (9–14 cm length, a size range typical of individuals at this site [8]) was added to the larger portion of each cage and allowed to graze for 20.5–22 h. This sampling duration was chosen based on prior observation of sediment color change after 24 h in the absence of sea cucumbers, potentially due to changes in the sediment microbiome (M.E.H. personal communication, per [8]).

On 18 June 2021, we collected: (i) sediment samples (surface 1–3 mm) from within the larger portion (grazed; in front of the cucumbers’ feeding tentacles) and inset portion (ungrazed) of each cage, (ii) fresh *H. atra* fecal pellets proximal to the sea cucumber’s anus, and (iii) sediment dissected from the sea cucumber’s foregut, midgut, and hindgut (GI tract) in the field (Fig. 1B). An enclosed sea cucumber escaped from one of the cages during the experiment, reducing our grazed samples to n = 9. On the same day we collected the samples from cages, we also collected samples external to, but nearby, the cages. These included sediments grazed by free-ranging (not caged) *H. atra*, fecal pellets proximal to the anus of free-ranging *H. atra*, and GI tract samples from nine free-ranging *H. atra* of similar size to those in the experimental population (Fig. 1C). GI tract samples were collected at the same time as paired fecal and sediment samples. Individual sea cucumbers were brought to the water surface, sliced longitudinally, and GI contents were sampled from the foregut, midgut, and hindgut of each specimen. Implements used in dissection and collection were rinsed thoroughly in ambient seawater between samplings.

All sample collection was completed between 1130–1300 h, with samples immediately preserved in RNA/DNA stabilizing buffer (25 mM sodium citrate, 10 mM EDTA and 70 g ammonium sulfate per 100 ml solution, pH 5.2) on ice in the field, and then stored at −40°C after return to the lab at 1700 h. Samples were shipped to Montana State University on dry ice and stored at −80°C until processing.

### DNA extraction

We estimated the abundance, taxonomy, and function of the sediment microbiome using DNA-based analyses. DNA was extracted from each sample by transferring 0.18–0.33 g sediment/feces (N = 99 total samples) into the bead beating tube of the Qiagen PowerSoil Pro kit (Qiagen, Hidalgen, Germany) with flame sterilized forceps. Extractions were performed following the manufacturer’s protocol, with extracted DNA quantified using a Qubit 4 Fluorometer (Thermo Fisher Scientific, Waltham, MA). An extraction blank (reagents only, no sample) was included in the workflow and used to evaluate contamination in the 16S rRNA gene amplification and sequencing process (below).

### Microbial abundance estimation—16S rRNA gene qPCR

To quantify impacts of sea cucumbers on microbial abundance, we used qPCR to count 16S rRNA gene copies per gram of fecal (n = 18), grazed sediment (n = 18), and ungrazed sediment (n = 9) samples. A standard curve relating 16S rRNA gene copy number to bacterial abundance was generated using DNA extracted from *Escherichia coli* K12. *E. coli* was grown to log phase OD_600_ of 0.6 at 4.2x10^8^ cells /ml [14], with each cell containing seven copies of the 16S rRNA gene [15]. Serial 1:10 dilutions from this culture were used to construct a standard curve relating 16S rRNA gene copies to the critical threshold (CT) of amplification. Aliquots (2 ml) from each step in the dilution series were pelleted by centrifuging at 16000 × g for 10 min. Supernatant was removed and cellular DNA extracted using the Qiagen Power Soil Pro with modifications. Briefly, 270 μl of lysis buffer and 270 μl of bead beating beads were added to the *E. coli* cell pellet and vortexed for 3 min. The full volume of the microcentrifuge tubes was transferred into the Qiagen bead beating tubes, an additional 530 μl of lysis buffer was added, and the tube was vortexed for 10 min. Subsequent extraction steps proceeded per manufacturer’s protocols. *E. coli* DNA was stored at 4°C, and all qPCR assays were completed within a week of extraction.

qPCR was performed using SYBR Green chemistry on a QuantStudio™ 5 Real-Time PCE system (Applied Biosystems, Waltham, MA) using the same primers as in the 16S rRNA gene sequencing described below [515 F (5′-GTGYCAGCMGCCGCGGTAA-3′; [16]) and 806R (5′-GGACTACNVGGGTWTCTAAT-3′; [17]); conditions in Supplementary Table 1]. All samples were run over 2 days with each sample run in triplicate and standards run in duplicate. Averaged technical replicate CT values for each sample were converted to copies of 16S rRNA genes based on the standard curve and then normalized to grams of sediment used in each sample’s DNA extraction.

#### Statistical testing and graphing

Our qPCR analyses sought to address the question of how sea cucumber grazing affected sediment microbial abundance. To answer this question, differences in mean gene copy number across sample types were compared with a linear mixed effects model (LME) accounting for the clustering of samples associated with the same sea cucumber and the effect of caging (Fixed: sample type [feces, grazed sediment…]; Random: cage status [in, out], cucumber replicate [1–18]), multiple comparisons were adjusted using Tukey–Kramer HSD, as implemented in the R packages lme4 (v1.1.34; [18]) and emmeans (v1.8.8; [19]). 16S rRNA gene copy number per gram of extracted sediment by sample type was visualized with violin plots as implemented in ggpirate (v0.1.2; [20]).

### Microbiome taxonomic analysis—16S rRNA gene amplicon sequencing

We used 16S rRNA gene amplicon sequencing to assess the impact of sea cucumber grazing on microbiome diversity and composition in grazed and ungrazed sediments and in sea cucumber foregut, midgut, hindgut, and fecal samples.

#### 16S rRNA gene sequencing

PCR amplification targeted the V4 region of the 16S rRNA gene using barcoded universal primers 515 F (5′-GTGYCAGCMGCCGCGGTAA-3′; [16]) and 806R (5′-GGACTACNVGGGTWTCTAAT-3′ [17];).

Amplicons were generated with the thermocycler protocol of [8] 95°C for 3 min, [1] 25 cycles of 95°C for 0:45 min, 55°C for 0:45 min, 72°C for 1:30 min, and [2] 72°C for 10 min (reaction components and volumes in Supplementary Table 1). Each amplicon was appended with unique 10 bp barcode identifiers for dual indexing (25 cycles). Amplicon products were then pooled in equimolar amounts and sequenced at Georgia Genomics and Bioinformatics Core on a MiSeq (Illumina Inc. San Diego, CA) using a paired-end 500 cycle kit with V2 chemistry, with 10% PhiX to increase read diversity.

#### Sequence processing

QIIME2 (v2022.8; [21] was used for amplicon analysis. Raw fastq files were imported into QIIME2, and reads were denoised, merged and filtered to remove chimeras using DADA2 [22] with read trimming parameters ‘--p-trim-left-f 20 --p-trim-left-r 20 --p-trunc-len-f 245 --p-trunc-len-r 245’ (quality control statistics in Supplementary Table 2). Taxonomy was assigned to each Amplicon Sequence Variant (ASV) using the Silva 138 515–806 database [23]. ASVs annotated as mitochondria, chloroplast, and known lab-specific contaminants (Supplementary Table 3) were removed, which removed all the ASVs in the extraction blank. To normalize sequencing effort, we rarified to 16 490 reads per sample without replacement, striking a balance between retaining as many samples as possible while removing samples with lower sequence yield (all excluded samples contained less than 9057 total reads; Supplementary Fig. 1). This filtering and rarefaction removed 11 (including the extraction blank) of the original 100 samples (composed of 99 cucumber-associated samples and 1 extraction blank).

#### Statistical testing and graphing

Analysis of 16S rRNA gene amplicon data addressed four questions: (i) How does sea cucumber grazing affect alpha diversity in sediment microbiomes? (ii) How does sea cucumber grazing affect beta diversity in sediment microbiomes? (iii) Are microbiome differences between grazed and ungrazed sediments linked to sediment microbiome change during passage through the sea cucumber GI tract? (iv) Which microbial taxa are enriched or depleted by sea cucumber grazing? To answer the first question, we generated alpha diversity metrics (Observed features, Faith’s phylogenetic diversity, Shannon diversity) in QIIME2 and tested for differences across sample types using a LME accounting for clustering of samples associated with the same sea cucumber and the effect of caging as implemented in the R packages lme4 (v1.1.34; [18]) and emmeans (v1.8.8; [19]. Alpha diversity was visualized with violin plots using ggpirate. To answer the second and third questions, we generated phylogenetic and abundance resolved distance matrices and tested clustering between samples with permutational multivariance analysis of variance (PERMANOVA) as implemented in R by adonis2 in vegan (v2.6.4; [24]). To account for cage effects, we restricted permutations between sample types by cage status [caged, uncaged] [25]. Significant (*P <* .05) PERMANOVA results warranted pairwise comparisons using the pairwise.adonis2 function in the package pairwiseAdonis (v0.4.1 [26]), maintaining the same cage status permutation scheme. Dispersion by sample type was compared between samples using permutational multivariate analysis of dispersion (PERMDISP) as implemented in the betadisper function in vegan, employing the same restricted permutations to control for cage status. Sample clustering was visualized with Non-Metric Multidimensional Scaling (NMDS) and Principal Coordinates Analysis as implemented in the R package vegan, and dispersion was visualized with violin plots using ggpirate. We visualized taxa shared between sample types using euler diagrams using the R package eulerr (v7.0.2; [27]). To answer the fourth question, taxa of interest were initially identified by qualitative examination and the percentage abundances of these taxa were center log ratio (CLR) transformed in the R package microbiome (v1.23.1; [28]) to satisfy the LME assumptions of equal variance and normal distribution, and visualized with pirate plots. Differences in transformed percentage abundance were assessed with LME. To visualize changes to composition, percentage abundances were plotted by sample as a stacked barplot using the R package ggplot2 (v3.4.3; [29]) or using CLR values on violin plots generated in ggpirate. In this analysis, LME model structure consisted of Fixed: sample type (feces, grazed sediment…); Random: cage status (in, out), cucumber replicate [1–18], multiple comparisons were adjusted using Tukey–Kramer HSD. In all cases, QIIME2 microbiome abundance matrices were imported into R (v4.3.1; [30]) using qiime2R (v0.99.6 [31]) and manipulated with phyloseq (v1.44.0; [28]) and tidyverse (v2.0.0; [32]).

### Microbiome metabolic potential—shotgun metagenomics

We used shotgun metagenomics to test how sea cucumber grazing affects the metabolic potential of the sediment microbiome. Both read-based (no assembly) and MAG-based approaches were used to compare ungrazed and grazed sediment metagenomes and to explore whether the grazed sediment microbiome resembles that of the fecal microbiome.

#### Metagenomic sequencing and quality control

Twenty (20) samples yielding the highest DNA concentrations were selected for shotgun sequencing, representing ungrazed (n = 6) and grazed (n = 7) sediment and fecal (n = 7) samples. Sequencing was done at the Harvard Bauer Core using 9 ng of DNA per sample. DNA was simultaneously fragmented and tagged with Illumina adapter sequences (tagmentation) using the Illumina DNA library preparation kit (Illumina Inc. San Diego, CA) creating ~350 bp inserts. Tagged inserts were enriched and 10 bp barcodes were appended with 12 cycles of amplification to allow multiplexing. After barcoding, libraries were purified with magnetic bead-based cleanup using Illumina Sample Purification Beads (Illumina Inc. San Diego, CA). Resulting libraries were assessed using a 4200 TapeStation (Agilent Technologies, Santa Clara, CA) and quantified by qPCR (Roche Sequencing, Indianapolis, IN). Libraries were pooled and sequenced (paired 150 bp reads) on one lane of a NovaSeq S4. Raw fastq files for the 20 metagenomes were filtered by quality score (Phred >25) and length (>100 bp) with TrimGalore! (v0.6.6; [33]), resulting in a cleaned dataset of 761 Gigabases (Gb), with average library size (forward + reverse reads) of 38.03 Gb and standard deviation of 12 Gb (Supplementary Table 4).

#### Read-based analysis

##### K-mer profiling

We used k-mer abundance patterns to agnostically identify differences in metagenomic composition among sample types. We used the program Simka (v1.5.1; [34]) to construct Bray-Curtis distance matrices based on 21-mer abundances in subsamples of reads (1,429 400 reads; accommodating the sample with the shallowest sequencing depth) from each metagenome. These matrices were used for the graphing described below.

##### 18S rRNA gene screening

We used the abundance of 18S rRNA genes in each metagenome to describe the abundance and diversity of microeukaryotes in grazed and ungrazed sediments. Reads matching the V4 region of 18S rRNA genes were identified and quantified using RiboTagger (v0.8.1; [35]) with the functions ribotagger.pl and biom.pl. Each matching sequence was assigned taxonomy by alignment against the NCBI nt database (as of October 2023) using BLASTn [36] with assignment based on the annotation of the top matching reference sequence with at-least phylum-level resolution.

##### Marker gene screening

To test the effect of sea cucumber grazing on microbial biogeochemical processes, we analyzed 59 marker genes of microbial metabolism. This database contained 39 genes used in prior studies by our group and others to screen for ecologically relevant metabolisms, including trace gas (e.g. methane) metabolism, dissimilatory sulfur and nitrogen metabolism, carbon fixation, photosynthesis, and aerobic respiration [37–39], and supplemented with an additional 20 marker genes for other biogeochemical or ecological strategies (e.g. sulfur reduction, secretion, flagellation). The latter 20 genes were obtained from UniProtKB [40] (Supplementary Table 5 lists all genes in our database). Gene relative abundances were estimated by aligning reads from each unassembled metagenome to each marker gene protein database with DIAMOND BLASTx (v2.0.15; [41]), applying a query coverage cutoff of 80% and amino acid identity cutoff of 50%. We estimated the proportion of community members encoding each gene by dividing the number of hits for each gene of interest by the gene’s average length in the database and again by the number of genomes (genome equivalents) in the metagenome as estimated in MicrobeCensus (v1.1.0 [42]), resulting in values as reads per kilobase genome equivalent (RPKG).

To assess the distribution of genome equivalents across the metagenomes to detect statistical outliers, we divided genome equivalents by sequencing effort (Mb) for each sample, and then fit a linear regression of GenomeEqivPerMb by Sample Type. After accounting for sample type, our data violated the assumptions of normality and equal variance, with one sample (‘06_in_wo_sediment’; ungrazed sediment) exhibiting a Cook’s Distance of 0.955 and identifying this sample as an influential outlier that could skew downstream statistical tests. Removing this sample resulted in normally distributed residuals and equal variances, satisfying the assumptions of the model (before and after diagnostic plots in Supplementary Fig. 2A, B). Further support for removing this sample came from the agnostic numerical classification of all samples based on the 21-mer frequencies, which showed this sample clustering separately from all other samples (NMDS and hierarchical clustering; Supplementary Fig. 3A, B). For these reasons, sample ‘06_in_wo_sediment’ was excluded from all subsequent metagenomic analyses.

#### Metagenome-assembled genome analysis

We used metagenome-assembled genome (MAGs) to assess the abundance of cyanobacteria in grazed and ungrazed sediments, requiring contig assembly, contig binning into MAGs, dereplication, and read mapping to estimate abundances. For contig assembly, reads were first normalized using BBnorm (https://sourceforge.net/projects/bbmap/), where kmer coverage was filtered to less than 100x and greater than 2x (“target = 100 mindepth = 2”). Metagenomes were individually assembled and then co-assembled into contigs using MEGAHIT (v1.2.9; [43]) with “meta-large” presets. Contigs were binned into MAGs using the MetaWRAP (v1.3.2; [44]) platform to map reads to the contigs using the BWA aligner (v0.7.17–1088; [45]). MetaWRAP was then used to generate MAGs with the binning programs MetaBAT 2 (v2:2.15; [46]) and MaxBin 2 (v2.2.7; [47]) utilizing contigs >2500 bp. MetaWRAP then identified the best quality MAGs from the two binning strategies based on contamination and completion estimates from CheckM (v1.1.3; [48]) and the “bin_refinement” module. Only MAGs with completeness >75% and contamination <10% (consistent with medium to high quality genomes; [49]) were retained for further analysis. MAGs were then dereplicated at 99% average nucleotide identity using dRep (v3.4.3; [50]) resulting in 154 high-to-medium quality dereplicated MAGs [49]. MAGs were assigned taxonomy using GTDB-Tk (v2.1.1; [51]), and relative abundance was estimated by mapping reads back to MAGs using BWA-mem in CoverM genome (v0.6.1; https://github.com/wwood/CoverM).

We focused our MAG-based analyses on cyanobacteria because they appeared to be markedly depleted after sea cucumber grazing in the 16S rRNA gene analyses. Only MAGs identified as cyanobacteria (n = 18; binning stats in Supplementary Table 6) were functionally annotated. We identified open reading frames (ORFs) with Prodigal [52] and annotated these genes using METABOLIC-G (v4.0; [53]; with criteria specified in Supplementary Table 5). We also screened these MAGs for the presence of cyanotoxin genes to assess the potential for cyanotoxin production. To do so, ORFs from each MAG (amino acid translation) were aligned against custom cyanotoxin biosynthesis protein databases (downloaded from UniProtKB and listed in Supplementary Table 5) using DIAMOND BLASTp in “ultra-sensitive” mode (v2.0.15; [41]), applying a query coverage cutoff of >80%, an amino acid identity cutoff of >40% and an e-value cutoff of <10^−50^.

#### Statistical testing and graphing

Our metagenomic comparisons addressed three questions: (i) Does sea cucumber grazing influence the composition of sediment metagenomes inferred from k-mer frequency? (ii) Does sea cucumber grazing influence the metabolic composition of sediment metagenomes inferred from marker genes? (iii) Does sea cucumber grazing influence the abundance of phototrophs?

To answer the first question, we used hierarchical clustering and NMDS to qualitatively assess sample clustering based on 21-mer-based distance matrices generated in Simka (see above). Bray-Curtis distances were square root-transformed and clustered using Ward’s cluster algorithm via the agnes function in the R package cluster (v2.1.4; [54]). Hierarchical clustering plots were generated with the base R package stats, and NMDS plots were generated with vegan (v2.6.4; [24]). To answer the second question, differences in mean RPKG across sample types were assessed with LME, which accounted for clustering due to sampling from the same sea cucumber and caged-uncaged status (Fixed: sample type [feces, grazed sediment…]; Random: cage status [in, out], cucumber replicate [1–18]), as implemented in the R packages lme4 (v1.1.34; [18]) and emmeans (v1.8.8; [19]). RPKG for each gene of interest was plotted with violin plots using ggpirate. To answer the third question, counts of 18S rRNA reads were normalized by dividing count values by the size of respective metagenome libraries, resulting in values as reads per megabase sequenced (RPM). MAG relative abundances were center log ratio (CLR)-transformed in the R package microbiome (v1.23.1; [28]) to satisfy the LME assumptions of equal variance and normal distribution. Differences in mean abundance for both metrics (18S RPM and MAG abundance) across sample types were assessed with LME (Fixed: sample type [feces, grazed sediment…], phototroph taxa [e.g. diatom, dinoflagellate, Xenococcaceae…], sample type * phototroph taxa; Random: cage status [in, out], cucumber replicate [1–18]), with abundance estimates visualized with violin plots using ggpirate.

## Results and discussion

Because prior research found that coral mortality increased following sea cucumber removal and was exacerbated by direct contact with sediment [9], we hypothesized that sea cucumber grazing restructured the sediment microbiome in a manner that lessened coral susceptibility to disease or other agents of mortality. We predicted that, in comparison to ungrazed sediment, grazed sediment would have: (i) lower microbial load, (ii) altered microbial community composition, and (iii) relative depletion of anaerobic metabolisms in sediment metagenomes due to mechanical aeration from sea cucumber grazing. Our results confirm predictions #1 and #2 but show that grazing is linked to sediment metabolic potential in an unexpected pattern. Compared to ungrazed sediments, sea cucumbers were associated with enrichment of anaerobic metabolisms and depletion of genes for cyanotoxin production. We discuss these results below.

### Sea cucumber grazing depletes sediment microbial abundance

**Figure 2:**
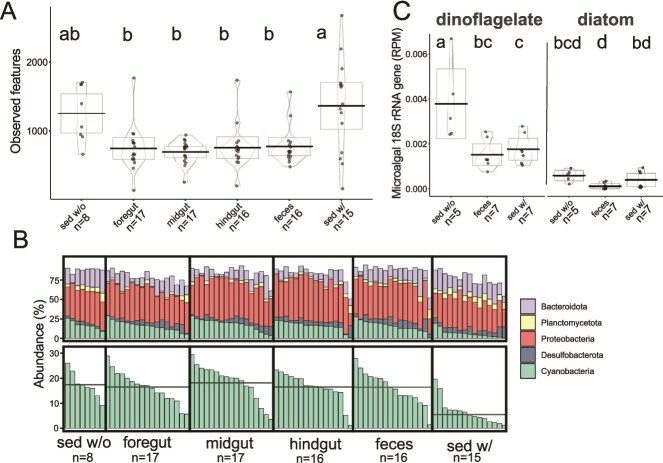
Sea cucumber grazing reshapes sediment microbiome diversity and depletes phototrophs. (A) Alpha diversity (observed features) in microbiomes of ungrazed (sed w/o) and grazed (sed w/) sediment compared to those of the sea cucumber gastrointestinal (GI) tract and feces. (B) Upper: Phylum-level community composition, including only phyla with minimum percentage abundances >0.25%. Lower: Same data as in upper graphs, but only including cyanobacteria; average percent abundances for each sample type shown as horizontal bar. Center log ratio (CLR)-transformed abundances are in Supplementary Fig. 8A. (C) Relative abundance of microalgal 18S rRNA genes in un-assembled metagenomes (metagenome data were generated only for sediments and fecal samples), with genes assigned phylum-level taxonomy based on top hits in the NCBI nt-nucleotide database. Normalized 18S rRNA gene abundances were calculated by dividing 18S rRNA gene reads by megabase of sequencing effort for each sample, resulting in reads per megabase (RPM). (A, C) Alpha diversity and normalized microalgal abundances presented as violin plots (mean as estimate of center; box bounded by 95% confidence interval; density estimator used to draw violin; observations as individual points). Dissimilar letters indicate *P <* .05 in pairwise comparison between groups, whereas sharing of any letter indicates *P >* .05 (*P* values were obtained from linear mixed effects model; Tukey–Kramer HSD).

The presence of *H. atra* reduced prokaryotic microbial load in coral reef sediment. The mean abundance of 16S rRNA genes in grazed sediments was ~75% lower than in ungrazed sediments, as estimated by qPCR (Fig. 1D; LME, *P <* .0001). Sea cucumber feces were similarly depleted in 16S rRNA gene copies, suggesting that cells are removed as sediment passes through the sea cucumber GI tract and that cell abundance does not immediately rebound after fecal deposition. This pattern was recapitulated in normalized extracted DNA, suggesting broader taxonomic depletion (Supplementary Fig. 4B). It is also possible that the physical churning of sediments by sea cucumbers allows for removal of microbes by water flow, facilitates grazing by other consumers, or alters the environment in a manner that suppresses microbial growth, although these mechanisms were not explored. We also acknowledge that variation in 16S rRNA gene copy number among prokaryotic taxa [55] could confound interpretation of qPCR data. However, for the purpose of this analysis, we assume that average gene copy number per cell is relatively consistent at the community level across samples, which is reasonable given that each sample is composed of hundreds or thousands of diverse taxa (Fig. 2A). Despite this caveat, our results suggest that sea cucumber presence substantially reduces sediment microbial load, as is consistent with studies showing a decrease in organics [56, 57] and microalgae [10] from sea cucumber feeding.

### Sea cucumber grazing reshapes sediment microbiome diversity

The presence of *H. atra* significantly influenced sediment microbiome diversity. Comparing 16S rRNA gene amplicons revealed significant differences in multiple beta diversity metrics in sediments with versus without sea cucumbers (Bray–Curtis and Weighted UniFrac; PERMANOVA *P <* .05; Supplementary Fig. 5B, C, E, F), suggesting that sea cucumber grazing changes the taxonomic composition of sediment microbiomes. However, community dispersion (PERMDISP, Bray-Curtis: *P =* .271 and Weighted UniFrac *P =* .101; Supplementary Fig. 5A, D), alpha diversity (LME, Observed features *P* = .837, Shannon diversity *P* = .744, Faith’s phylogenetic diversity *P* = .937; Fig. 2A, Supplementary Fig. 6A, B) and evenness (LME, Simpson’s Evenness *P* = .330; Supplementary Fig. 6C) did not vary significantly between sediments with versus without sea cucumbers. The results suggest that the presence of sea cucumbers predominantly affects the taxonomic composition – but not richness or evenness – of the sediment microbiome, potentially via selective removal or enrichment of microbial taxa during passage of sediment through the GI tract.

Comparison of microbiomes in ungrazed and grazed sediment and digesta demonstrates that sediment microbiomes change during passage through the sea cucumber GI tract. Microbiome composition varied significantly at each stage of sediment digestion, from before [ungrazed, sed w/o], to during [foregut, midgut, hindgut, feces], and after [grazed, sed w/]) grazing (Bray–Curtis and Weighted UniFrac; PERMANOVA, *P <* .05; Supplementary Fig. 5B, C, E, F). Microbiome alpha diversity also changed with digestion, being relatively high in ungrazed sediment, immediately decreasing in the foregut and staying depressed throughout the GI tract and feces, then rebounding in the grazed sediment to a level indistinguishable from ungrazed sediments (LME, *P <* .05, Fig. 2A: Observed features, Supplementary Fig. 6B: Faith’s phylogenetic diversity). Evenness remained steady over most of the course of digestion (LME, Simpson’s Evenness *P >* .273; Supplementary Fig. 6C), except that cucumber grazed sediments have more even representation of taxa compared to the foregut microbiome (LME, Simpson’s Evenness *P* = .051; Supplementary Fig. 6C). A marked decrease in microbial diversity in the sea cucumber foregut has been previously observed, consistent with the sea cucumber GI microbiome being generally less diverse than that of the surrounding sediments [58, 59]. This may be due to efficient enzymatic degradation of sediment microbes [58] or selective feeding, although *H. atra* has been shown to exhibit less specific feeding than other observed Holothurids [60]). Broadly consistent with our results, prior work with *Holothuria leucospilota* showed lower microbiome diversity in sea cucumber feces compared to surrounding sediments [58].

Both beta and alpha diversity differed significantly between grazed sediments (sed/ w) and sediments conglomerated into fecal pellets (Fig. 2A, Supplementary Figs. 5B, C, E, F, & 6B). Furthermore, of 11 071 prokaryotic ASVs detected across feces and grazed sediment samples, only 23% are shared in both sample types, whereas 20% and 57% are unique to feces and grazed sediment, respectively (Supplementary Fig. 7).

These results suggest that passage of sediments through sea cucumbers influences, but does not fully explain, microbiome diversity in grazed sediments. The microbiome in grazed sediments is distinct from that of ungrazed sediments but does not simply reflect the sea cucumber GI microbiome spread across the reef. This pattern is consistent with recent work on another *Holothuria* species, which identified a gut microbiome distinct from that of surrounding sediments both in taxonomic composition and in having lower alpha diversity, suggestive of removal of microbial taxa during digestion or adaptation to the intestinal niche [58]. These and our results suggest that activity of sea cucumbers likely influences the sediment microbiome through a combination of mechanisms, potentially including: (i) selective consumption of microbes whose metabolisms or behavior disproportionately shape sediment microbiome composition (e.g. oxygen-producing phototrophs, bacterivorous protists), (ii) alteration of the diversity and concentration of chemical substrates (e.g. particulate organic carbon) supporting microbial metabolism in sediments, (iii) physical disruption of the sediment biofilm to facilitate access by other consumers or microbial dispersal out of sediments, and (iv) release of a fecal microbiome that serves as a distinct starting point (inoculum) for successional regrowth of the sediment microbiome. Our analysis of microbiome gene content (below) provides preliminary insight into some of these hypotheses.

### Sea cucumber grazing significantly depletes phototrophs

A diverse set of microbial taxa differed in relative abundance between ungrazed and grazed sediments, with Cyanobacteria as the Phylum showing the largest variation. The CLR-transformed average relative abundance of cyanobacterial 16S rRNA gene amplicons was significantly higher in ungrazed (17.2%) compared to grazed sediment (6.5%; LME, *P <* .05; Fig. 2B, Supplementary Fig. 8A), with sequences matching Microcystaceae being particularly depleted in the presence of sea cucumbers (from 3.1% to 1.2% without versus with grazing, respectively; LME, *P <* .05; Supplementary Fig. 9). This decline in amplicon-based relative abundance was observed alongside the qPCR-based decline (mean 75%) in total 16S rRNA genes per gram of sediment under grazing, suggesting that the absolute numerical abundance of cyanobacteria decreased by an estimated 90% under grazing. A substantial depletion was also observed in metagenomic sequences mapped to cyanobacterial MAGs (Supplementary Fig. 8B). In contrast to expectations, the relative abundance of cyanobacterial amplicons was significantly higher in feces compared to grazed sediments (Fig. 2B). This suggests that cyanobacterial depletion from grazed sediments is not due solely to prokaryote removal by digestion (Fig. 1D), but rather to some other mechanism, potentially including changes in substrate availability (e.g. sea cucumbers competitively consuming nutrients [56]) or physical reworking of the grazed sediment environment preventing cyanobacterial establishment [56].

The relative abundance of eukaryotic microalgae also decreased under sea cucumber grazing, based on the frequency of 18S rRNA genes in metagenomes (Fig. 2C, Supplementary Fig. 10). Changes to dinoflagellate abundances were the most obvious (Supplementary Fig. 10), with 18S rRNA genes being ~ 50% less common in metagenomes from both grazed sediments and feces compared to metagenomes from ungrazed sediments (LME, *P <* .05). Although less abundant across all samples compared to dinoflagellate genes, diatom 18S rRNA genes were also depleted (~50%) in feces compared to ungrazed sediment metagenomes, consistent with literature suggesting that *H. atra* digests eukaryotic microalgae [10] and indeed contains a microbiome specifically adapted for digestion of microalgae and bacteria [58]. However, diatom gene abundances did not significantly differ between grazed and ungrazed sediment metagenomes (Fig. 2C). These patterns suggest that even in the presence of sea cucumber grazing, microalgal taxa such as diatoms may rebound quickly in reef sediments, whereas other taxa such as dinoflagellates remain depleted.

These taxonomic data link sea cucumber grazing to declines in the relative abundance of both cyanobacterial and eukaryotic phototrophs in sediment microbiomes. Given the qPCR results reported above, the biomass of cyanobacteria in surface reef sediments presumably also declines substantially under grazing. Eukaryotic phototroph biomass likely also declines but was not measured here. This removal of photosynthetic microbes is consistent with tank experiments showing decreased algal growth with versus without sea cucumbers (e.g. [61]) and field observations showing an increase in green or rust-colored pigmentation [8] and presumed cyanobacterial mat growth after sea cucumber exclusion [9, 56].

### Sea cucumber removal of phototrophs may influence coral health

**Figure 3:**
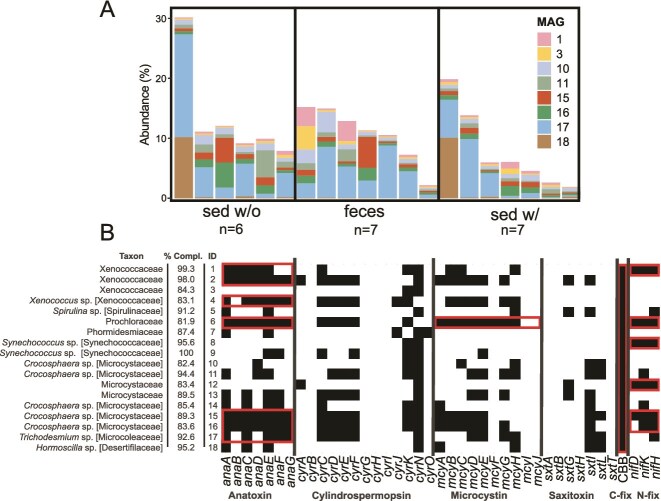
Abundant, high-quality cyanobacterial metagenome-assembled genomes (MAGs) encode genes for cyanotoxin biosynthesis. (A) Percentage abundance of the most abundant cyanobacterial MAGs. Abundance was estimated by first recovering MAGs from sea cucumber feces and sediment samples using individual and co-assembly approaches, then percentage abundances of cyanobacterial MAGs were estimated by mapping metagenomic reads to all dereplicated MAGs (99% ANI, >75% completion, <10% contamination) and plotted the most abundant MAGs (max abundance >3%). (B) Cyanobacterial MAGs contain diverse cyanotoxin biosynthesis genes and carbon and nitrogen fixation genes. Cyanotoxin genes were identified by querying (via DIAMOND BLASTp) open reading frames (amino acid translation) to custom databases for each gene of interest (see Supplementary Table 5). The thresholds for positive gene detection were > 40% sequence identity, >80% query coverage, and < 10^−50^ e-value. MAGs possessing all but 1–2 genes for any queried cyanotoxin gene cluster are indicated with a red bounding box. Carbon and nitrogen fixation genes were identified via hidden Markov models as implemented in METABOLIC-G. MAG binning statistics are in Supplementary Table 6. Taxon, finest taxonomic resolution available—Brackets used to specify family if genus is available; ID, unique cyanobacterial MAG identifier; % Compl., estimated MAG percentage completion; *anaA-G*, anatoxin; *cyrA-K,N,O*, cylindrospermopsin toxin; *mcyA-J*, microcystin toxin; *sxtA,B,G,H,I,L,T*, saxitoxin; CBB, Calvin-Benson-Basham cycle genes; *nifD,K,H*, nitrogenase; C-fix, carbon fixation; N-fix, nitrogen fixation.

Corals may be harmed if the absence of sea cucumbers leads to an increased abundance of sediment phototrophs. Cyanobacteria, in particular, are known for producing compounds allelopathic to animals. Cyanotoxins such as microcystin, as well as other cyanobacteria-derived metabolites, have been found in cyanobacteria-dominated coral lesions and suggested to contribute to the progression of black band disease (BBD) [62, 63]. Here, several known cyanotoxin-producing families, including the Microcystaceae, Phormidiaceae, and Cyanobacteriaceae [64–66], were present in our 16S rRNA gene datasets in the absence of sea cucumbers (Supplementary Fig. 9). To follow-up on this association, we searched our 18 cyanobacterial MAGs (Fig. 3A) for evidence of cyanotoxin genes. Many cyanotoxins are produced through non-ribosomal protein synthesis pathways encoded by well-described biosynthetic gene clusters [67–71], with the presence of these genes being an indicator of cyanotoxins in the environment [72, 73].

We detected several near-complete cyanotoxin gene clusters in diverse cyanobacterial MAGs from feces and sediment samples (Fig. 3B). Six of these MAGs (2 *Crocosphaera* sp. [Microcystaceae], 2 Xenococcaceae, 1 Prochloraceae, and 1 *Trichodesmium* sp. [Microcoleaceae]) contain all or nearly all the queried gene cluster for synthesis of anatoxin (*anaA-G*). Anatoxin is neurotoxic to humans and many vertebrates [74], and recent literature has shown this alkaloid toxin is variably toxic to aquatic invertebrates [75, 76]. The anatoxin-encoding Prochloraceae MAG also contains most of the genes for synthesizing microcystin (*mcyA-H*), lacking only *mcyI* and *mcyJ*. Gene clusters for the cyanotoxins cylindrospermopsin and saxitoxin were also present but were incomplete in all MAGs (Fig. 3B).

Cyanobacteria may also contribute to diminished coral health through mechanisms that do not involve known cyanotoxins. For example, cyanobacteria of the genus *Pleurocapsa* (Xenococcaceae) are not known for cyanotoxin production but have been associated with low recruitment of acroporid and pocilloporid coral larvae [77, 78]. Here, *Pleurocapsa* 16S rRNA genes were more abundant in 16S rRNA gene datasets when sea cucumbers were absent. However, a mechanism(s) by which *Pleurocapsa* may interact (negatively or positively) with corals has not been confirmed.

Coral health may also be affected by other phototrophs detected in our datasets, including dinoflagellates. Dinoflagellate blooms have been linked to coral death (e.g. [79]), and the potential for dinoflagellates to harm corals is well known in coral aquaculture (F.J.S. personal communication). Dinoflagellate blooms likely harm corals primarily by influencing oxygen, nutrient, or light availability. However, dinoflagellates also produce toxins, some of which can affect coral physiology, e.g. by altering calcium ion flux across membranes [80]. Though our sediment metagenomes did not contain dinoflagellate contigs sufficient for detecting toxin genes, we observed a relative depletion of dinoflagellate 18S rRNA gene fragments from metagenomes when sea cucumbers are present (Fig. 2C). Collectively, our results raise the hypothesis that sea cucumbers benefit corals by removing potentially harmful cyanobacteria, dinoflagellates, or other phototrophs.

### Sea cucumber grazing reshapes microbiome metabolic potential, enriching for genes of anaerobic metabolisms

**Figure 4:**
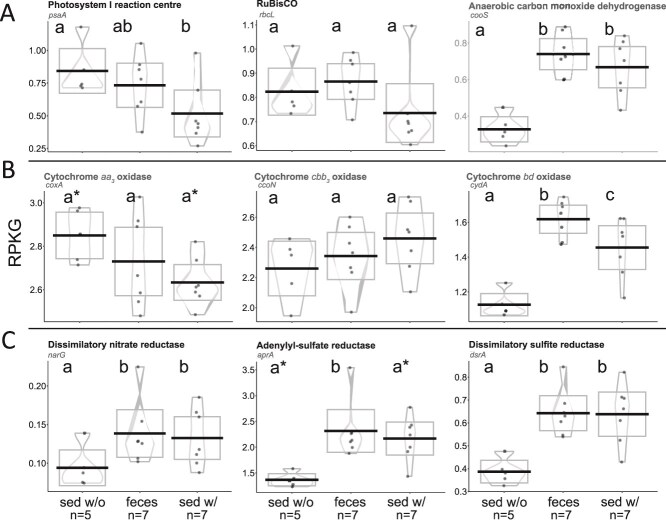
Microbiome metabolic potential differs between ungrazed sediment (sed w/o) versus sediment grazed (sed w/ and feces) by sea cucumbers, with anaerobic metabolisms enriched in grazed sediments. Relative abundance of marker genes shown as reads per kilobase genome equivalent (RPKG). (A) Photosynthesis and carbon fixation genes, showing depletion of phototrophs and enrichment in anaerobic carbon fixation genes. *psaA*, subunit of photosystem I reaction center (photosynthesis); *rbcL*, ribulose-1,5-bisphosphate carboxylase/oxygenase (RuBisCO) large subunit (Calvin-Benson-Basham carbon fixation), *cooS*, carbon monoxide dehydrogenase (anaerobic Wood Ljundahl carbon fixation). (B) Cytochrome oxidase genes, showing enrichment of high O_2_ affinity (*cbb_3_* and bd -type) oxidase genes with sea cucumber grazing. (C) Oxygen-sensitive reductive energy metabolism genes, showing increase in potential use of non-O_2_ electron acceptors with sea cucumber grazing. *narG*, dissimilatory nitrate reductase alpha subunit (nitrate reduction); *aprA*, adenylyl-sulfate reductase alpha subunit (sulfur reduction); *dsrA*, dissimilatory sulfite reductase alpha subunit (sulfur reduction). Gene abundance presented as violin plots (mean as estimate of center; box bounded by 95% confidence interval; density estimator used to draw violin; observations as individual points). Dissimilar letters indicate *P <* .05 in pairwise comparison between groups, whereas sharing of any letter indicates *P >* .05; shared letters with an asterisk indicates *P <* .10 between contrasts (*P* values were obtained from linear mixed effects model; Tukey–Kramer HSD).

Sea cucumbers may also alter the metabolic properties of the sediment microbiome, potentially in ways that influence coral health. To explore this potential, we analyzed sediment metagenomes for marker genes of biogeochemically relevant metabolisms. Following our 16S and 18S gene observations of sea cucumbers depleting phototrophs, we also observed that sea cucumber grazing significantly reduced the relative abundance of genes for oxygenic photosynthesis, including *psaA* and *psbA* encoding components of photosystem I (PSI) and photosystem II (PSII) respectively, responsible for the splitting of water and production of oxygen during light-dependent reactions (Fig. 4A, Supplementary Fig. 11C). The relative abundance of *rbcL*, encoding the large subunit of ribulose-1,5-bisphosphate carboxylase/oxygenase responsible for Calvin cycle-based carbon fixation did not vary. Similarly, *nifH* encoding nitrogenase for nitrogen fixation also decreased in abundance under grazing, most likely due to removal of diazotrophic cyanobacteria, examples of which were detectable in the MAG data (Fig. 3B, Supplementary Fig. 11B). This depletion of genes associated with photosynthesis or phototrophs is consistent with the taxonomic trends described above and predictions that sea cucumber grazing removes primary producers.

In contrast to our expectations, sea cucumber grazing resulted in a significant enrichment of diverse genes linked to anaerobic or microaerophilic metabolism. Concurrent with removal of oxygenic phototrophs, marker genes of the anaerobic Wood-Ljungdahl (*cooS* encoding carbon monoxide dehydrogenase Fig. 4A; *acsB* encoding acetyl-CoA synthase, Supplementary Fig. 11D) and reductive-TCA (*aclB* encoding ATP citrate synthase, Supplementary Fig. 11D) carbon fixation pathways became significantly more abundant. Markers of dissimilatory anaerobic metabolism also increased significantly. These included genes of denitrification (*narG* encoding nitrate reductase, Fig. 4C; *nirS* and *norB* encoding nitrite and nitric oxide reductase, respectively, Supplementary Fig. 11B), anaerobic ammonium oxidation (anammox; *hzsA* encoding hydrazine synthase; Supplementary Fig. 11A), and sulfur reduction (*dsrA* encoding dissimilatory sulfite reductase; Fig. 4C; *aprA* encoding adenylyl sulfate reductase, *asrA* encoding anaerobic sulfite reductase, *sat* encoding sulfate adenylyltransferase, Supplementary Fig. 11B). Genes encoding terminal oxidases with differing affinities for oxygen also changed in abundance consistent with a decrease in oxygen availability under grazing (Fig. 4B). In contrast to ungrazed sediments, sediments under grazing were depleted of genes encoding lower O_2_ affinity *aa_3_*-type cytochrome c oxidase genes typically associated with respiration under normoxic conditions. Rather, grazed sediments were enriched in genes for higher affinity *cbb_3_*- and *bd*-type cytochrome oxidases, with this enrichment being significant and largest for *bd* variants. Both *cbb_3_* and *bd* oxidases are associated with microorganisms conducting microaerobic respiration under limited oxygen availability [81–83].

The representation of oxygen-sensitive marker genes in fecal metagenomes differed from that of grazed or ungrazed sediment, but not consistently for all genes (Fig. 4B, Supplementary Fig. 11). For some genes, their relative abundance in feces was similar to, or in some cases indistinguishable from (e.g. *narG, aprA, dsrA, cooS*), values in grazed sediment. However, other genes were intermediate in abundance between values in grazed and ungrazed sediment (e.g. *coxA, psaA, ccoN*), whereas still others were significantly elevated in feces compared to both sediment types (e.g. *napA, cydA, asrA, sat;*  Supplementary Fig. 11B, D, C). These complex functional gene patterns are consistent with the taxonomic trends reported above, suggesting that grazed sediment microbiomes may be influenced by, but are nonetheless distinct from, fecal microbiomes.

Collectively, the marker gene data indicate that sea cucumber grazing alters the functional potential of marine sediments, enriching anaerobic or microaerobic metabolisms suggestive of a decrease in local oxygen concentrations. This pattern is seemingly not consistent with the hypothesis that sea cucumber bioturbation aerates reef sediments. The pattern is instead consistent with 1) the observed removal of phototrophs by grazing, which would likely remove a local oxygen source (oxygenic photosynthesis) from sediment biofilms, and 2) addition of gut-associated and low-oxygen adapted microbial communities to sediment via sea cucumber defecation. Though the data provide support for both mechanisms, other mechanisms may also be contributing. For example, it is likely that grazed sediment represents some combination of ungrazed sediment grains and sediment grains held together by fecal casing/mucus, which may serve to limit oxygen diffusion from overlying waters relative to ungrazed sediment. However, we stress that we did not measure sediment oxygen concentration in this study but that future measurements of oxygen are essential for validating the predictions based solely on gene data, as well as the potential mechanisms contributing to oxygen changes.

## Conclusions

Sea cucumber grazing significantly transforms the abundance and biochemical potential of reef sediment microbiomes. Sea cucumber presence decreases the abundances of all bacteria, particularly oxygenic phototrophs, and by extension the abundance of oxygen and cyanotoxin production pathways. Though we initially predicted that sea cucumber grazing, like that of many other bioturbating freshwater and marine invertebrates (e.g. freshwater oligochaetes [84], marine crabs [85], marine polychaetes [86], wetland oligochaetes and insects [87]), would increase oxygen flux into sediments, our data suggest that the presence of sea cucumbers instead results in an elevated relative potential for sediment anaerobic processes (including sulfide production from sulfate reduction or desulfurylation or the removal of oxygen for coral respiration (e.g. [12, 88]), at least within the timeframe of our *in situ* experiments. Collectively, these results suggest sea cucumbers influence the potential for cyanotoxin, oxygen, and anaerobic metabolite production, all of which have potential to affect coral survival.

However, we caution that our results reflect metabolic potential. Measurements of sediment microbial activity and metabolite and oxygen concentration were not obtained but could help identify potential chemical mechanisms by which the sea cucumber-sediment interaction influences coral health. Indeed, it is possible that the absolute concentration of metabolites associated with anaerobiosis (e.g. sulfide) may still decline under grazing, given the coinciding and substantial reduction in sediment microbial load. Chemical measurements are justified given that prior work (e.g. [9]) has not identified a pathogen(s) explaining the increased coral mortality in the absence of sea cucumbers. The current study suggests that metabolites of cyanobacterial or microalgal origin might be particularly likely to vary with sea cucumber abundance, given the pronounced effect of sea cucumber grazing on sediment phototrophs, and therefore potential candidates for having health effects on corals.

Sea cucumbers exert diverse, significant, and potentially cascading effects on both microbial and macroorganismal (e.g. coral) components of coral reefs. The mechanisms by which these effects are connected remain uncertain but are likely linked to a transformation of sediment microbial biogeochemistry under sea cucumber grazing. Identifying these mechanisms may be critical for mitigating the detrimental effects of historic and ongoing overharvesting of sea cucumbers from reefs worldwide.

## Supplementary Material

sea_cucumber_microbiome_figures_supplementary_wraf088

sea_cucumber_microbiome_tables_supplementary_wraf088

## Data Availability

All sequence data (16S rRNA gene amplicons, metagenomes, and cyanobacterial MAGs) were deposited at the National Center for Biotechnology Information under BioProject PRJNA1136881.
