# Supplementary material for: Sea cucumber grazing linked to enrichment of anaerobic microbial metabolisms in coral reef sediments
Source: ISME J. 2025 May 3;19(1):wraf088. doi: 10.1093/ismejo/wraf088 (PMC12201990; doi:10.1093/ismejo/wraf088)
Supplement: sea_cucumber_microbiome_figures_supplementary_wraf088 [file sea_cucumber_microbiome_figures_supplementary_wraf088.pdf]

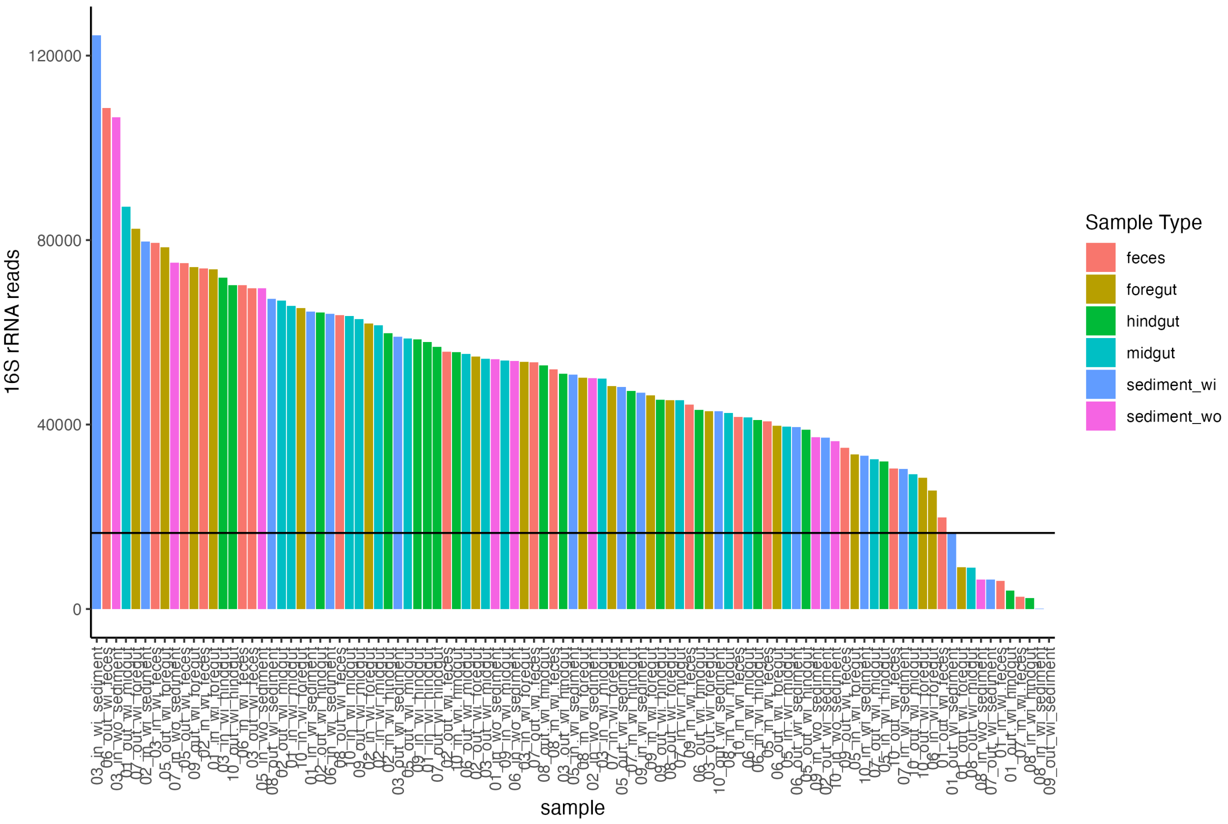

Supplementary Figure 1: Rarefaction of 16S rRNA amplicons. 16S rRNA reads per sample were normalized by rarefaction to a sequencing depth of 16,490 reads per sample (horizontal line) removing 9 samples. Sample type, categorical classification of samples from collection; feces, *Holothuria atra* fecal samples collected proximal to the sea cucumber's anus; foregut, *H. atra* foregut samples; midgut, *H. atra* midgut samples; hindgut, *H. atra* hindgut samples; sediment_wi, sediment samples collected proximal to *H. atra* mouthparts; sediment_wo, sediment collected from within the caged *H. atra* exclosure.

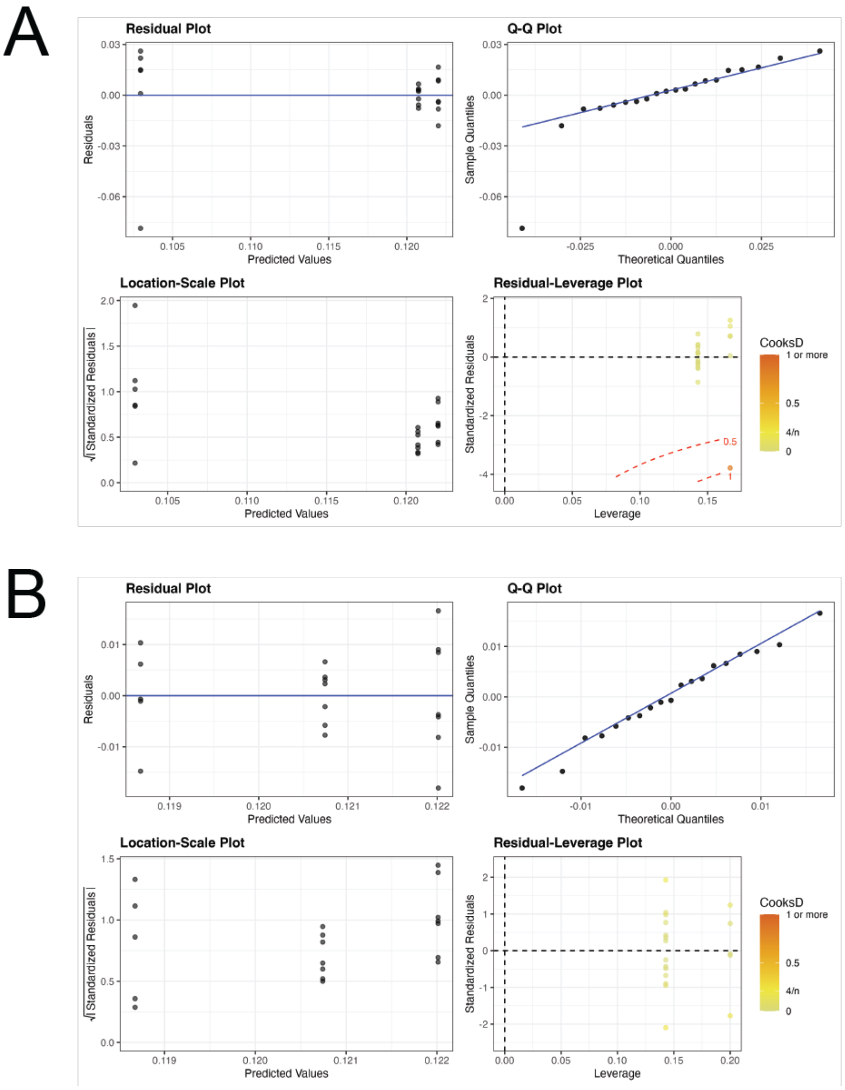

Supplementary Figure 2: Diagnostic plots for linear model of genome equivalents per megabase (Mb) by sample type [lm(GenomeEqivPerMb~sample_type)]. **A)** Plots with sample 06_in_wo_sediment included. In the residual vs. fitted plot there is strong evidence against the assumption of equal variance, in the QQ plot, there is strong evidence against the assumption of normal distribution, and in the Residual-Leverage plot, there is a likely influential outlier with a Cooks distance of 0.955. **B)** Plots when sample 06_in_wo_sediment is excluded. In the residual vs. fitted plot there is weak evidence against the assumption of equal variance, in the QQ plot, there is weak evidence against the assumption of normal distribution, and in the Residual-Leverage plot, there are no influential outliers.

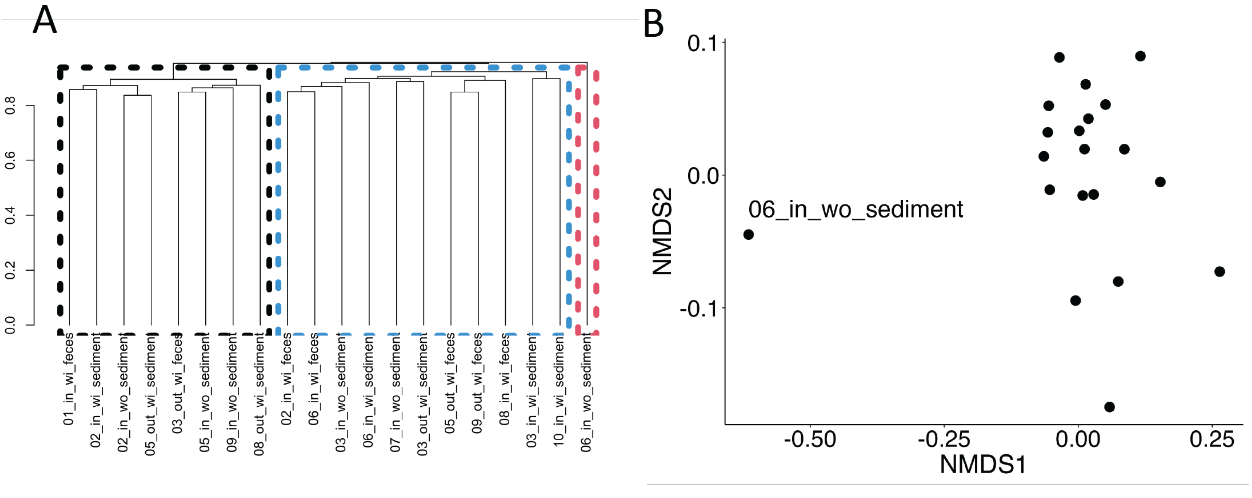

Supplementary Figure 3: <u>Ordination of grazed and ungrazed sediments using k-mer based distance matrices from unbinned metagenomes demonstrates that sample 06_in_wo_sediment deviates from all other samples</u>. **A)** hierarchical clustering of square root transformed Bray-Curtis distance matrices derived from 21-mer abundances using Ward's Clustering Algorithm as implemented in the agnes function in the cluster package in R. **B)** Non-Metric Multidimensional Scaling (NMDS) plots of non-metric Bray-Curtis distance matrices based on 21-mer abundances of available samples.

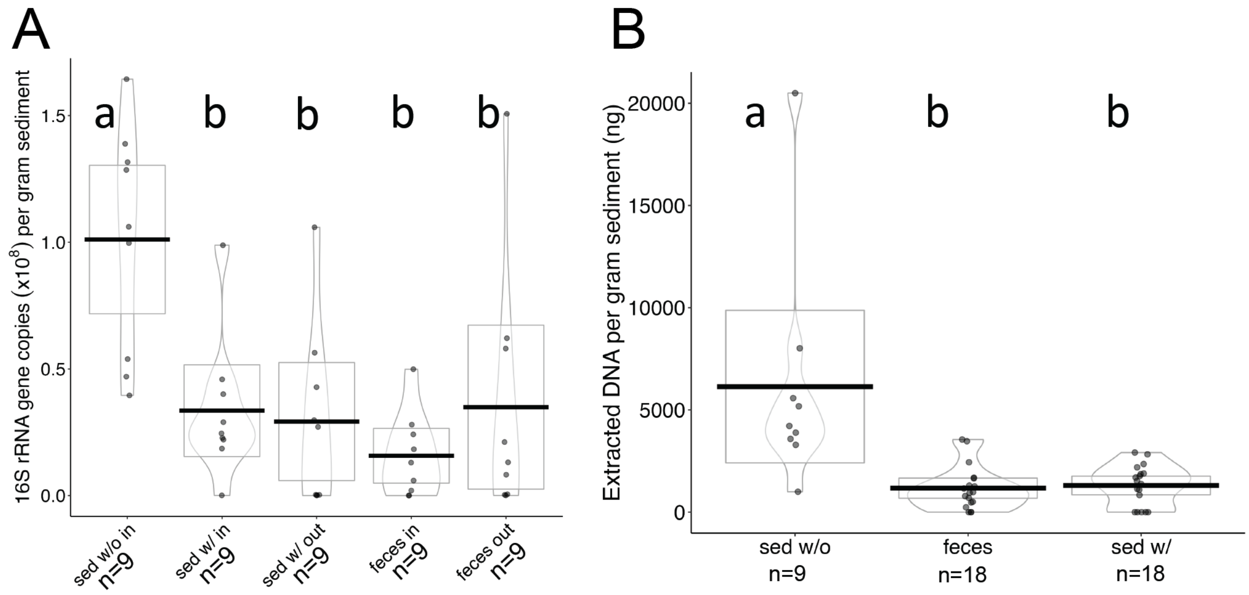

Supplementary Figure 4: Estimating microbial abundances before and after cucumber grazing shows that cucumber grazing reduces proxy measures for microbial load. **A)** Quantitative PCR of 16S rRNA gene copies normalized to grams of sediment used in DNA extraction. **B)** Extracted DNA per gram of sediment from each sample type normalized to grams of sediment used in DNA extraction. Quantitative measures presented as violin plots (mean as estimate of center, box bounded by 95% Confidence interval (CI), density estimator to draw violin, observations as individual points). Dissimilar letters indicate $P<0.01$ between contrasts *(P* values were obtained from linear mixed effects model; Tukey-Kramer HSD).

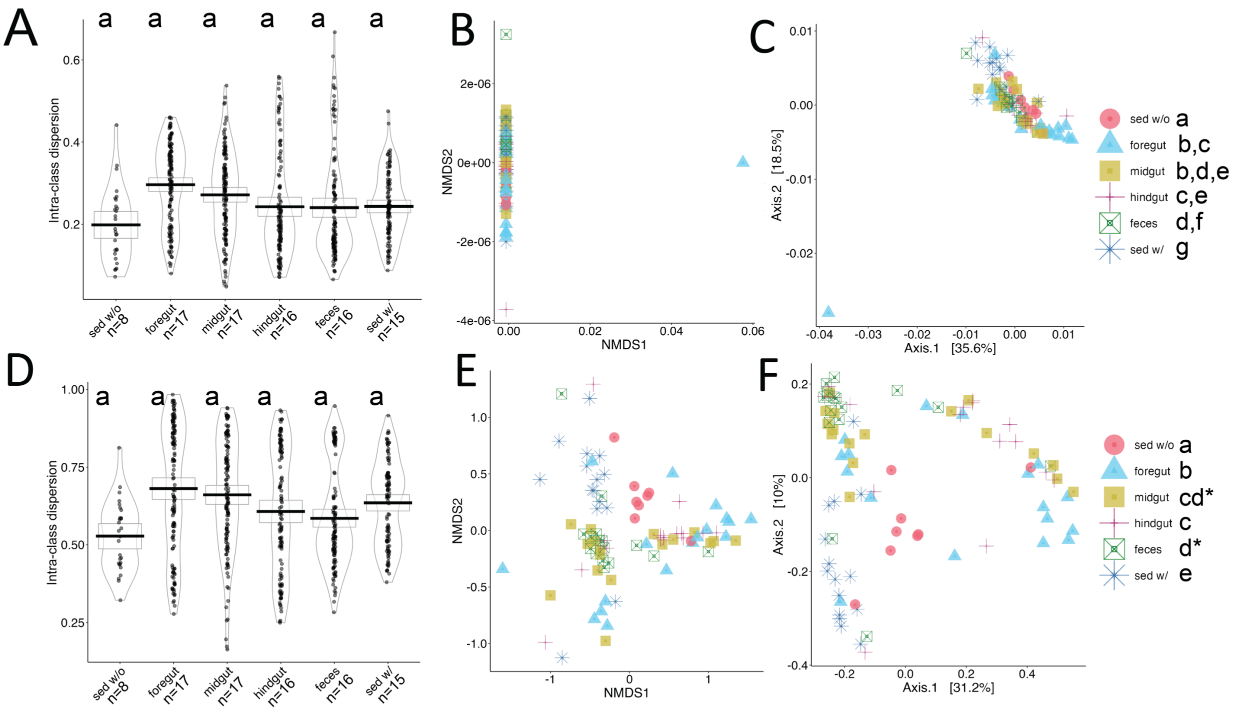

Supplementary Figure 5: Beta diversity of sediment samples before, during and after digestion from 16S rRNA gene data show that sea cucumber grazing alters community composition, but not dispersion. Displaying **A,D**) intra-class dispersion and **B,E**) Non-Metric Multidimensional Scaling (NMDS) and **C,F)** Principal Coordinates Analysis (PCoA) of communities using **A-C**) Weighted UniFrac and **D-F**) Bray-Curtis distance matrices. Intra-class dispersion presented as violin plots (mean as estimate of center, box bounded by 95% Confidence interval (CI), density estimator to draw violin, observations as individual points). Shared letters indicate $P>0.10$ between contrasts (Permutational Analyses of Multivariate Dispersions, PERMDISP; permutations restricted by cage-status to control for cage-status). Statistically detectable differences in community composition presented as dissimilar letters on the respective keys, with shared letters with an asterisk indicating $P<0.10$ between contrasts ($P<0.05$; permutational multivariate analysis of variance, PERMANOVA; permutations restricted by cage-status to control for cage-status).

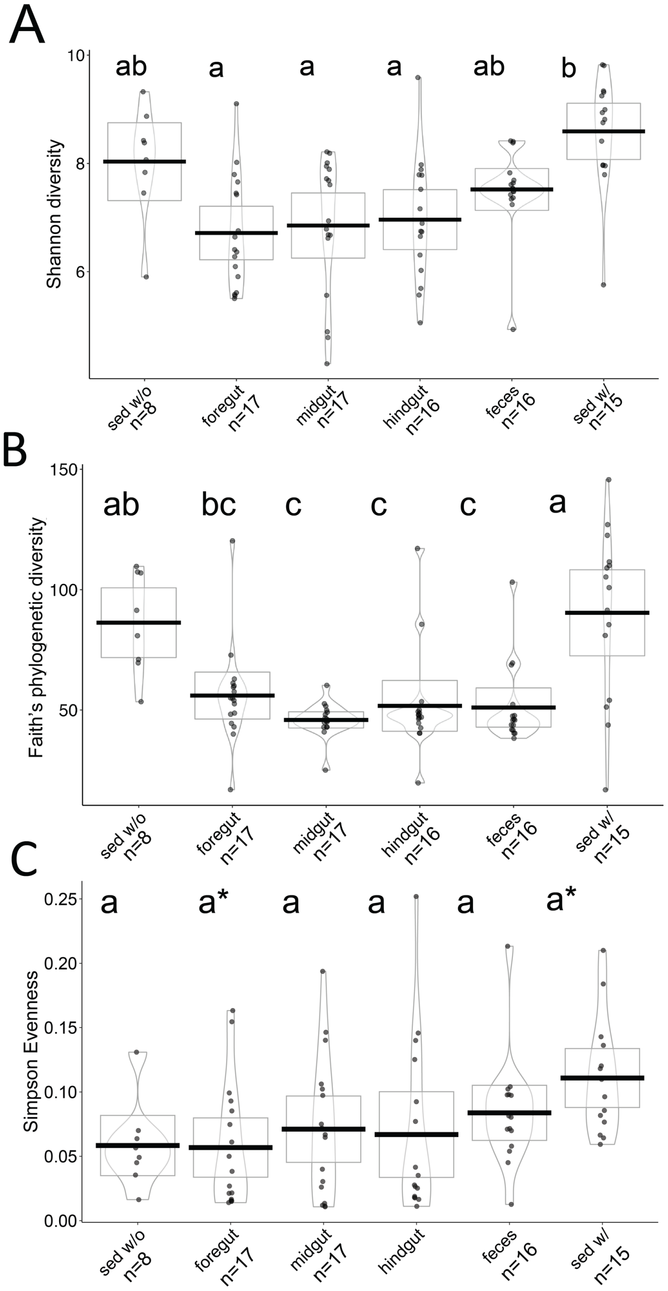

Supplementary Figure 6: <u>Alpha diversity of sediment samples before, during and after sediment digestion from 16S rRNA gene data shows that cucumber GI tract and external sediment differs</u>. **A)** Shannon's Diversity, **B)** Faith's phylogenetic diversity, **C)** Simpson's Evenness. Alpha diversity metrics presented as violin plots (mean as estimate of center, box bounded by 95% Confidence interval (CI), density estimator to draw violin, observations as individual points). Dissimilar letters indicate *P*<0.05 between contrasts, shared letters with an asterisk indicates *P*<0.10 between contrasts (*P* values were obtained from linear mixed effects model; Tukey-Kramer HSD).

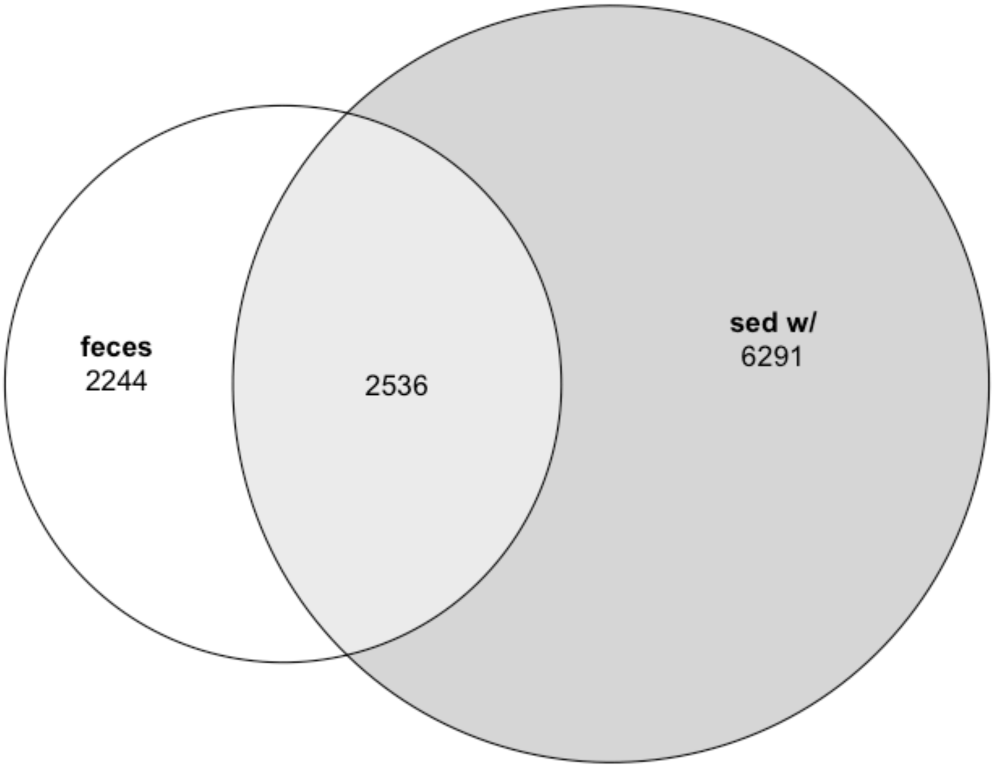

Supplementary Figure 7: 16S rRNA gene amplicon sequence variants (ASVs) shared between sea cucumber feces and sea cucumber-grazed sediments. The area is plotted proportionally to the number of ASVs in each group.

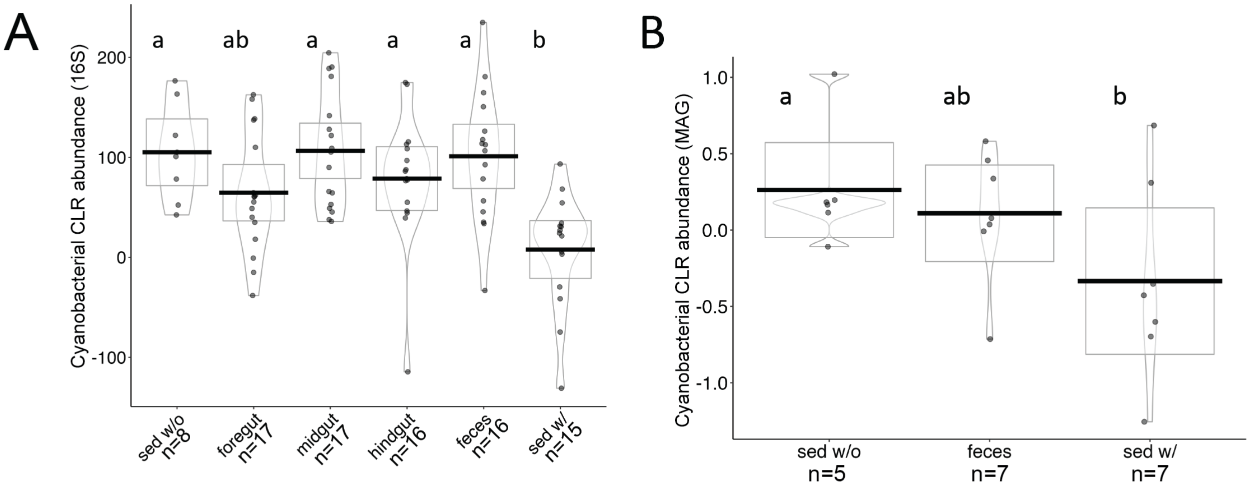

Supplementary Figure 8: <u>Depletion of cyanobacterial transformed relative abundances in grazed sediments.</u> Inferred from **A)** 16S rRNA genes and **B)** metagenome assembled genomes (MAGs). **A)** 16S rRNA gene abundances were estimated from the percent of cyanobacterial reads relative to the total reads. **B)** Cyanobacterial MAG abundances were estimated by mapping metagenomic reads to all dereplicated MAGs (99% ANI, >75% completion, <10% contamination). Abundance presented as violin plots (mean as estimate of center, box bounded by 95% Confidence interval (CI), density estimator to draw violin, observations as individual points). Dissimilar letters indicate $P<0.05$ between contrasts *(P* values were obtained from linear mixed effects model; Tukey-Kramer HSD). Cyanobacterial percentage abundances were center-log ratio (CLR) transformed to satisfy mixed effects model assumptions of normal distribution and equal variance.

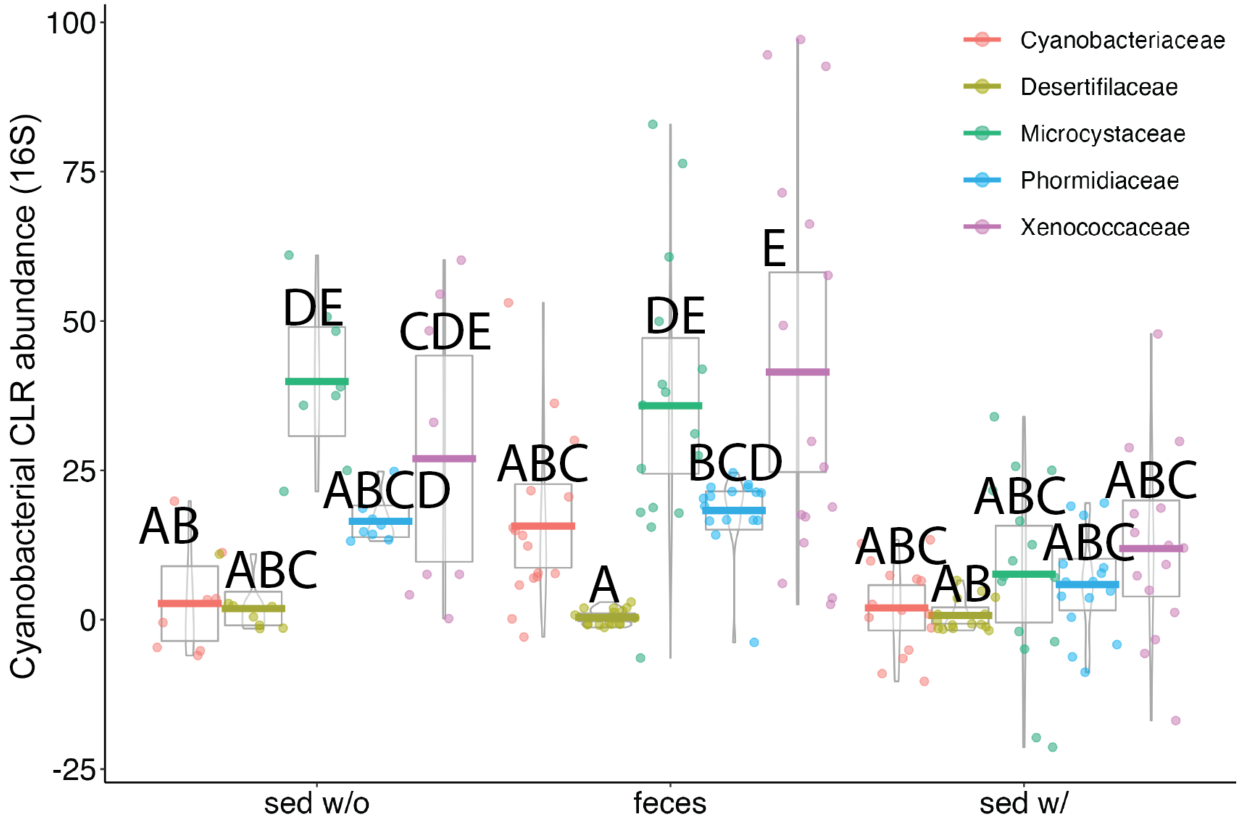

Supplementary Figure 9: <u>Changes in cyanobacterial family abundances before and after grazing from 16S rRNA gene data</u>. Only cyanobacterial families with maximum percentage abundances of >1.5% are shown. Cyanobacterial center-log ratio (CLR) transformed abundances presented as violin plots (mean as estimate of center, box bounded by 95% Confidence interval (CI), density estimator to draw violin, observations as individual points). Dissimilar letters indicate $P<0.05$ between contrasts *(P* values were obtained from linear mixed effects model; Tukey-Kramer HSD). CLR transform was implemented to meet mixed effects model assumptions of normal distribution and equal variance.

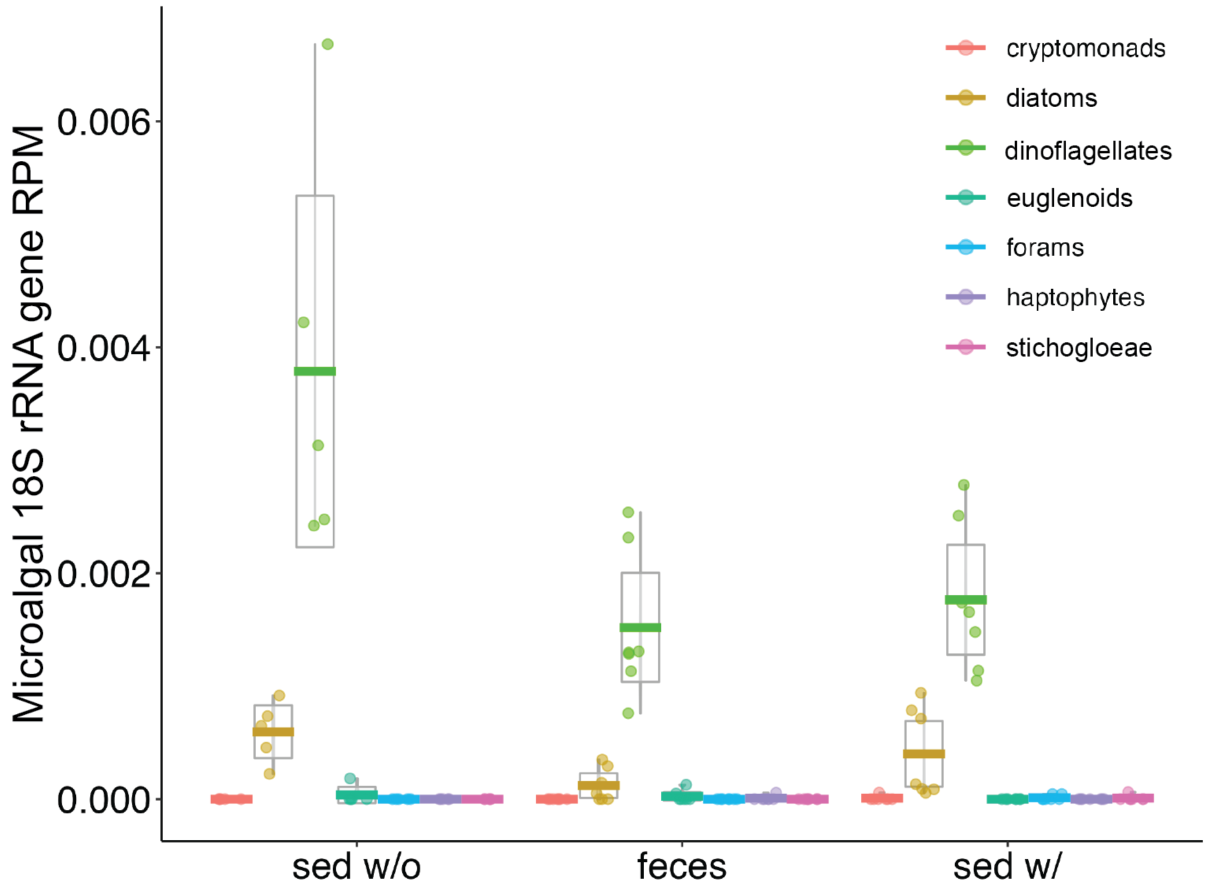

Supplementary Figure 10: Normalized microalgal abundance of 18S rRNA genes before and after cucumber grazing. 18S rRNA counts were derived from metagenome sequencing Normalized abundance (reads per megabase; RPM) presented as violin plots (mean as estimate of center, box bounded by 95% Confidence interval (CI), density estimator to draw violin, observations as individual points).

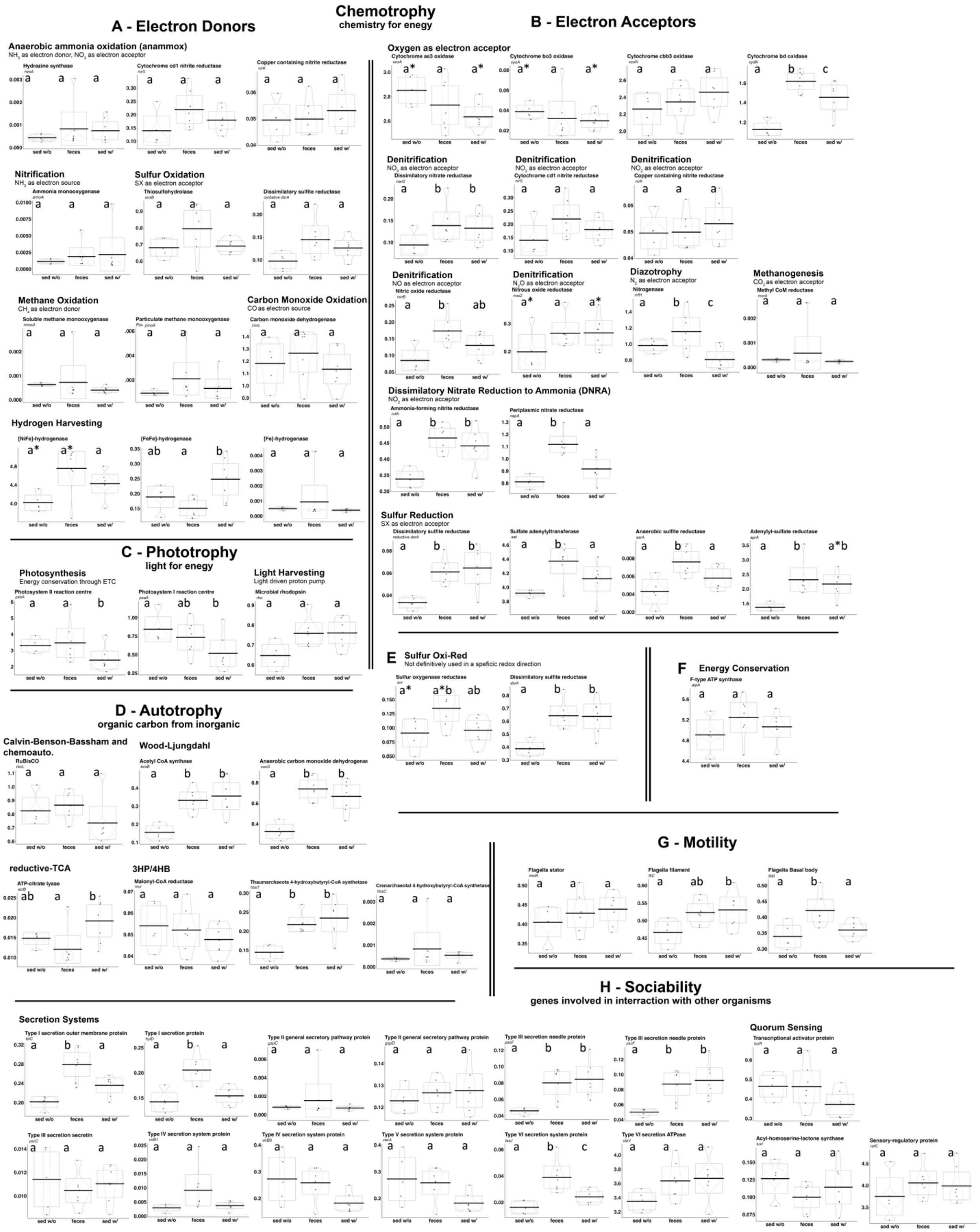

Supplementary Figure 11: Inferred metabolic potential of the microbial community from unassembled metagenomes. Relativized abundance of genes as reads per kilobase genome equivalent (RPKG) of diagnostic genes for ungrazed sediment (sed w/o), feces, grazed sediment

(sed w/). Genes grouped by functional categories: **A) Electron donors.** Anaerobic ammonia oxidation (anammox): *hzsA*, Hydrazine synthetase; *nirS*, cytochrome cd1 nitrite reductase; *nirK*, copper containing nitrite reductase. Nitrification: *amoA*, ammonia monooxygenase. Sulfur oxidation: *soxB*, Thiosulfohydrolase; oxidative *dsrA*, dissimilatory sulfite reductase. Carbon monoxide oxidation: *coxL*, carbon monoxide dehydrogenase. Methane oxidation: *mmoA*, soluable methane monooxygenase; *pmoA*, particulate methane monooxygenase. Hydrogen harvesting: [NiFe]-Hydrogenase; [FeFe]-hydrogenase; [Fe]-hydrogenase. **B) Electron acceptors**. Oxygen reduction: *coxA*, cytochrome aa_3 oxidase; *cyoA*, cytochrome bo_3 oxidase; *ccoN*, cytochrome cbb_3 oxidase; *cydA*, cytochrome *bd* oxidase. Denitrification: *narG*, dissimilatory nitrate reductase; *nirS*, cytochrome cd1 nitrite reductase; *nirK*, copper containing nitrite reductase; *norB*, nitric oxide reductase; *nosZ*, nitrous oxide reductase. Diazotrophy: *nifH*, nitrogenase. Methanogenesis: *mcrA*, methyl CoM reductase. Dissimilatory nitrate reduction to ammonia: *nrfA*, ammonia-forming nitrite reductase; *napA*, periplasmic nitrate reductase. Sulfur reduction: Reductive *dsrA*, dissimilatory sulfite reductase; *sat*, sulfate adenylytransferase; *asrA*, anaerobic sulfite reductase; *aprA*, adenyly-sulfate reductase. **C) Phototrophy**. Photosynthesis: *psbA*, Photosystem II reaction center; *psaA*, Photosystem I reaction center. Light driven proton pump: *rho*, microbial rhodhopsin. **D) Autotrophy**. Calvin-Benson-Bassham and Chemolithoautotrophy: *rbcL*, RuBisCo. Wood-Ljungdahl: *acsB*, Acetyl CoA Synthetase; *cooS*, Anaerobic carbon monoxide dehydrogenase. reductive-TCA: *aclB*, ATP Citrate-lyase; *mcr*. 3HB/4HB: Malonyl CoA Reductase; *hbsT*, Thaumarchaeota 4-hydroxybutyryl-CoA Synthetase; *hbsC*, Crenarchaeotal 4-hydroxybutyryl-CoA Synthetase. **E) Sulfur oxidation/ reduction**: *sor*, sulfur oxygenase reductase; *dsrA*, dissimilatory sulfite reductase. **F) Energy conservation**. *atpA*, F-type ATP synthase. **G)** Motility. *motA*, flagella stator; *fliC*, flagella filament; *fliM*, flagella basal body. **H) Sociability**. Secretion Systems: *tolC*, type I secretion system outer membrane protein; *hylD*, type I secretion system; *gspC*, type II general secretory pathway protein; *gspD*, type II general secretory pathway protein; *pscF*, type III secretion needle protein; *yscF*, type III secretion needle protein; *yscC*, type III secretion secretin; *virB1*, type IV secretion system protein; *virB5*, type IV secretion system protein; *vacA*, type V secretion system protein; *tssJ*, type VI secretion system protein; *clpV*, type VI secretion system ATPase. Quorum Sensing: *luxR*, Transcriptional activator protein; *luxI*, Acyl-homoserine-lactone synthase; *rpfC*, Sensory-regulatory protein. Gene abundance presented as violin plots (mean as estimate of center, box bounded by 95% Confidence interval (CI), density estimator to draw violin, observations as individual points). Dissimilar letters indicate $P<0.05$ between contrasts, shared letters with an asterisk indicates $P<0.10$ between contrasts (Mixed Effects Model, fixed: sample type; random: cucumber samples collected nearest from, cage/uncaged status; Tukey-Kramer HSD).
